# Fairness overrides reputation: the importance of fairness considerations in altruistic cooperation

**DOI:** 10.3389/fnhum.2013.00252

**Published:** 2013-06-07

**Authors:** Şule Güney, Ben R. Newell

**Affiliations:** School of Psychology, University of New South WalesSydney, NSW, Australia

**Keywords:** altruistic cooperation, mini ultimatum game, fairness, reputation building, future interaction, intentions

## Abstract

Behavioral findings in several strategic games indicate that people punish others if they think they are being treated unequally, even at the cost of minimizing their own material payoff. We investigated the primary driving force behind such non-self-regarding behavior, so-called, altruistic cooperation. In all of our studies, a mini ultimatum game was played either one-shot (in Experiment 1a and 1b) or repeatedly (Experiment 2), and rejections of inequitable distribution were taken as a measure of altruistic cooperation. In Experiment 1a, we replicated previous findings indicating that the key mechanism contributing to the emergence of altruistic cooperation is fairness considerations. In Experiment 1b, we delved into the relative importance of two aspects of fairness considerations (i.e., outcome fairness and intentions) and showed that both aspects were effective in determining the level of altruistic cooperation, with the contribution of intentions being more important. In Experiment 2, we investigated the effect of the opportunity for reputation building and future interaction on altruistic cooperation. We found that these factors became influential only when fairness considerations were weakened, particularly, as a result of the removal of the possible intentions behind an offer.

## Introduction

Human altruistic cooperation presents a puzzle from the perspectives of both the standard economic models of the “self-interested actor” and the evolutionary models of the “self-regarding individual” because it involves some characteristics that are difficult to reconcile with the predictions of standard game theoretical and evolutionary analyses. One form of altruistic cooperation is to reward cooperators (i.e., costly rewarding) and to punish norm violators (i.e., costly punishment) at a personal cost, even though the probability that this cost will be repaid (either by third parties or by that specific agent in the future) is very low (Gintis et al., 2003)^1^.

Evidence for the existence of altruistic cooperation largely comes from laboratory experiments in which the respective behavioral pattern has been observed through economic games. One of the best-known economic games used to demonstrate altruistic cooperation, particularly costly punishment, is the Ultimatum Game (UG) (Güth et al., 1982). In this game two players are presented with a sum of money; one of them is assigned to the role of Proposer while the other one to the role of Responder. The Proposer is asked to offer any portion of the money to the Responder. If the Responder accepts the amount offered, the money is distributed in accordance with the proposal. If the Responder rejects the offer, both get nothing.

According to standard economic theory of self-interest, a rational Proposer offers the minimum possible amount, and a rational Responder never rejects any amount unless it is zero (Binmore, 2007). The underlying assumption in this prediction is that both parties care only about how much money they get. However, the vast majority of experimental studies has shown that the modal offers by the Proposers lie between 40–50% of the total amount and the Responders frequently reject offers below 25% (Güth et al., 1982; Roth, 1995; Henrich et al., 2005). This pattern of results has been replicated cross-culturally (Henrich et al., 2005) and shown to be robust with large stakes (Cameron, 1999).

The experiments reported here aimed to investigate the differential contributions of fairness considerations and perceived opportunity of reputation building (RB)/future interaction to the emergence of costly punishment as a form of altruistic cooperation in experimental contexts.

Some researchers argue that the underlying mechanism of such non self-regarding behaviors in the UG (i.e., high offers by the Proposers and frequent rejections by the Responders) is not only to get as much money as possible, but also to maintain fairness norms among players (Fehr and Gachter, 2002; Gintis et al., 2003). In other words, the players have a preference for fairness, along with the preference for material benefits (Falk et al., 2008). In fact, the motivation behind the Proposers' high offers can be explained with or without the involvement of fairness considerations: they simply may not want to offer an amount that can be easily turned down by the Responder, so they are willing to distribute the money in a relatively fair way. Thus, the Proposers' main concern still might be getting as much as possible in the end, rather than treating the Responders fairly (Declerck et al., 2009). However, for Responders, the role of fairness concerns is more apparent and must be stronger because they seem to accept ending up with nothing rather than being treated unfairly. Even though the Responders could have been better off by accepting any amount offered, they prefer to punish the Proposer's unfairness, at a cost to themselves. This pattern of response indicates that the Responders engage in costly punishment in response to the unfairness of the *outcome* proposed by the Proposer^2^.

A special version of UG has been used to demonstrate how much the Responders care about (un)fair *intentions* of the Proposers. The structure of the so-called Mini UG (see Table 1) is the same as the standard UG, with an exception: the Proposer is again asked to distribute an amount of money but unlike the standard UG, only in one of two ways. Both players participate in four consecutive Mini UGs, and throughout all these games one way of distribution is always fixed while the alternative distribution is always different across games. The fixed distribution is a relatively inequitable one (i.e., the Proposer can take $8 for himself, and offer $2 to the Responder, see Table 1).

**Table 1:** **General structure of Mini Ultimatum Games**.

Mini Ultimatum Games^*^							
	**(5/5) Game**		**(2/8) Game**		**(10/0) Game**		**(8/2) Game**	
Possible distributions	(8/2)	(5/5)	(8/2)	(2/8)	(8/2)	(10/0)	(8/2)	(8/2)
Perceived fairness of the (8/2) distribution	Unfair		Reasonably unfair^**^		Fair		Neutral	

* *The numbers in the parentheses denote how much the Proposer could get/how much the Responder could get*.

** *The Proposer seems to have an excuse for offering the more inequitable distribution (8/2), because otherwise he would be unfair to himself [i.e., by offering the (2/8) distribution, he would give 8 to the Responder, and take 2 himself]*.

However, the available alternative distribution varies in terms of the outcome fairness, sometimes yielding a more equitable outcome (i.e., the Proposer can take $5 for himself, and offer $5 to the Responder, see Table 1), and sometimes yielding an even more unequal outcome (i.e., the Proposer can take $10 for himself, and offer $0 to the Responder, see Table 1). Under the standard assumptions, rejection rates for the fixed distribution (8/2) are expected to be the same regardless of its alternatives, as its monetary value stays unchanged across games (Falk et al., 2003a). However, this particular distribution was rejected much more frequently when the Proposer intentionally ignored the more equitable alternative distribution [i.e., the (5/5) distribution] than when he ignored the more unequal alternative distribution [i.e., the (10/0) distribution] (Falk et al., 2003a; Sutter, 2007). Thus, the rejection decisions made by the Responders seem not to be determined by the absolute amount of the offer (i.e., $2), but by whether the offer is seen as relatively unfair [i.e., in comparison to (5/5) split] or fair [i.e., in comparison to (10/0) split]. [See Table 1 for the perceived fairness of the fixed distribution (8/2) across four games]. These findings indicate that the Responders punish the unfairness of the Proposers by rejecting an amount of money in one case and appreciate the fairness of the Proposer by accepting the very same amount in another case. It has been argued therefore that fairness considerations must be the underlying motive behind altruistic cooperation, especially in the context of costly punishment (Fehr and Gachter, 2002; Gintis et al., 2003).

Although the importance of fairness considerations in such bargaining games has been widely accepted, the real reasons for altruistic cooperation (i.e., the Responders' rejection/acceptance behaviors in the UG) have been a source of much debate (Declerck et al., 2009). As mentioned earlier, by rejecting a non-zero offer, the Responders seem to engage in actions that are opposite to their self-interest, in order to maintain the fairness norms between parties. Thus, fairness considerations seem to override the self-regarding/rational motives.

Confidence in such a conclusion mainly comes from the two critical features of the above-mentioned experiments: identities and the decision histories of both players are kept hidden (i.e., anonymous) and they will never meet again in another round (i.e., one-shot encounter). Anonymous and one-shot encounters eliminate the possibility of reputation building (henceforth, RB) and future interaction (henceforth, FI) respectively, as potential sources of this seemingly fairness-driven behavior (Fehr and Fischbacher, 2003). Involvement of *any* of these possibilities -either RB *or* FI-would be especially critical in this context because the costly behavior obtained in these experiments could then be explained within the boundaries of self-regarding motives: it is rational and adaptive to reject unfair offers if the possibility of re-encountering the same game partner in the future is high enough *or* if the possibility of building a reputation among other players is at stake. The underlying reason for this claim is that rejecting unfair offers protects the player from being offered with unequal distributions by the same game partner in the future or by third parties, and thus this behavior serves the player's self-interest (Burnham and Johnson, 2005; Hagen and Hammerstein, 2006).

This argument goes further in the direction that people engage in altruistic cooperation in one-shot and anonymous encounters simply because they confuse the experimental settings with the more familiar environments where interactions are normally repeated and non-anonymous (Burnham and Johnson, 2005). In fact, the participants might still be responding to implicit cues suggesting that future interaction is possible or that their reputation is at stake. One finding that supports this interpretation is that the presence of eyespots on the computer desktop, which triggers the sense that participants are being watched, leads to increased generosity in another money allocation game (Haley and Fessler, 2005). Some other studies suggest that even the perception of being involved in a situation where FI and RB is possible triggers altruistic cooperation in one-shot, and anonymously played economic games (Kiyonari et al., 2000). Thus, behaving in an altruistically cooperative manner in the UGs might not solely result from the concern for the maintenance of fairness norms, but from the mis-perceived opportunity of RB and FI (Haley and Fessler, 2005; Bateson et al., 2006).

In the set of studies reported here, we aimed to investigate how important these two factors, namely fairness considerations (in Experiment 1a and 1b) and the possibility of RB and FI (in Experiment 2), are in the emergence of altruistic cooperation in general and costly punishment in particular. Experiments 1a and 1b were designed to understand the role of fairness considerations in costly punishment. Note that, as pointed out previously, fairness considerations have two major aspects, one being related to *outcomes* (Fehr and Schmidt, 1999; Bolton and Ockenfels, 2000), and the other to *intentions* (Rabin, 1993; Dufwenberg and Kirchsteiger, 2004). Although, several economic models have been developed with a specific focus on outcome fairness (Fehr and Schmidt, 1999; Bolton and Ockenfels, 2000), empirical evidence suggests that intentions are just as important as outcomes (Blount, 1995; Falk et al., 2008) for the maintenance of fairness norms. This is why we thought it was necessary to incorporate both of these important aspects of fairness consideration into our investigation and hence we used the Mini UG, instead of the standard UG, in all of our experiments.

Previous studies have already established the importance of intentions behind an action (i.e., offer) in the Mini UG: the (8/2) distribution is rejected at different levels depending on whether the alternative distributions are perceived as fair or not (i.e., highest rejections observed when the alternative was more equitable). However, findings diverge in terms of rejection rates of the (8/2) distribution when the alternative distribution was more inequitable. More specifically, 9% of the Responders rejected the (8/2) distribution in the (10/0) game in Falk et al.'s (2003a) study whereas almost 28% rejected it in Sutter's (2007) study. Considering these differences in previous findings, Experiment 1a was conducted to re-establish the basic phenomenon observed in the Mini UG (presented in Table 1). We found it preferable to observe the standard rate of rejections in all Mini UGs in our own subject pool first, in order to provide a standardized baseline before incorporating the subsequent manipulations (Experiments 1b, 2) (and potential implications to be drawn from these manipulations).

Experiment 1b was designed to clarify the relative impact of these two aspects of fairness considerations in the Mini UG. Two special features of this specific version of UG enable us to separate the effect of intentions from that of outcomes (Falk et al., 2008): the Proposer has two available options to distribute the allocated money, with one option always being more equitable or yielding a fairer outcome (compared to the other option). Importantly, the choice of one distribution over the other is under the Proposer's full control [except for the (8/2) game, see Table 1]. In order to differentiate the effect of intentions from the effect of outcome fairness, we removed the latter feature from the Mini UG and thus made any potential attribution of intentions impossible, but kept the former and thus made the evaluation of outcome fairness possible. If the rejections of the (8/2) distribution are primarily determined in response to the (unfair) intentions of the Proposer, then we should not obtain any differences in these rejections rates across the games because the intentions behind the offers are not assessable. However, it has been already shown that the Responders react to the fairness of outcomes as well (Blount, 1995; Falk et al., 2008). Thus, different rejection rates among different Mini UGs were expected but this manipulation would enable us to examine if these differences would be as strong as those observed in Experiment 1a where intentions were assessable.

In Experiment 2, we aimed to understand the combined effect of the *real* possibility of RB and FI in the Mini UG. The main reason for testing the combined effect of RB and FI was that in the above-mentioned studies demonstrating fairness driven responses (i.e., different rates of rejection of an inequitable distribution across Mini UGs), the features of “one-shot-interaction” and “anonymity” are inseparable. Therefore, it is difficult to identify whether the obtained responses could actually be the product of the (mis)perception of one-shot encounters as repeated (and thus players behave as if re-encountering the same game partner in the future is possible in order to maximize their material pay-offs) *or* that of the (mis)perception of anonymous encounters as non-anonymous (and thus Responders behave as if building a reputation among other players is possible in order to maximize their material pay-offs). Thus, incorporation of both possibilities of RB and FI through repeated and non-anonymous game play would make the two previously mentioned explanations (fairness-driven responses via one-shot/anonymous encounter vs. self-regarding responses via misperceived one-shot/anonymous encounter) commensurable. A second and even a more explicit reason was that the possibility of RB and FI are highly interrelated (i.e., repeated encounters with the same partner, by default, bring along the opportunity of building reputation as each player would know what the other player has done so far).

We predicted that if the main reason behind the rejections in one-shot and anonymously played games is the *misperceived* possibility of RB or FI, then an increase in the level of altruistic cooperation should be expected when the actual possibility of RB and FI is added to the context. Although such an additional effect of the possibility of RB and FI has not been investigated in the Mini UG, there are two main reasons for expecting such an increase. First, the importance given to equality is expected to be elevated (Rottemberg, 2008) because the equality norm (i.e., distributing the allocated money evenly) is strengthened in presence of the possibility of RB and FI (Hertel et al., 2002). Second and more importantly, the sanctions inflicted upon the unfairness of a game partner through altruistic cooperation might be considered as an effective tool for maximizing future gains (Kiyonari et al., 2000).

The structure of Mini UG allows us to examine how the possibility of RB and FI, along with the fairness concerns, contributes to the Responders' rejections especially when costly punishment is expected to take place (i.e., when the alternative offer yielded a more equitable distribution). In addition, in the Mini UG, there is one special game [the (8/2) game, see Table 1] in which the Proposer has no choice, but to offer the fixed amount. This particular case would enable us to detect the sole effect of the possibility of RB and FI on the Responders' decisions when an unequal distribution was offered without any (un)fair intentions of the Proposer involved. For all these reasons, to the best of our knowledge, Experiment 2 is the first attempt to understand the effect of the real possibility of RB and FI on costly punishment, particularly in the presence and absence of Proposer's intentions.

## Experiment 1A

We expected the rejection rate of the (8/2) distribution to be different across different Mini UGs. More specifically, the highest rejection rate was expected to be in the (5/5) game. In addition we expected to find statistically significant differences between the rejection rates of the (8/2) distribution in the (5/5) and the (10/0) games.

### Method

#### Participants

Fifty first year psychology students (*M* age = 19.5, 36 female) at UNSW participated in the experiment as a part of their course requirement, and were informed that they would be paid, contingent on the outcome of their choices. UNSW Human Research Ethics Advisory Panel approved the study.

#### Procedure

There were 10 experimental sessions in total, and five participants were tested at a time in each experimental session. Participants were seated in separate rooms and their identities were kept hidden throughout the whole experiment. All participants played the Mini UG as the Responders since our main interest was to see whether we would be able to replicate the choice pattern of the Responders obtained in previous studies (i.e., Falk et al., 2003a). However, each participant was told that only one participant in each group of five would be assigned to the Responder role and that the rest would be playing as Proposers. This procedure made them believe that the offer in each game would come from an actual but different participant (Proposer) rather than from the computer. The offers made by the computer mimicked the actual rate of proposals offered by real Proposers in the study of Falk et al. (2003a). For instance, in that study, the (8/2) distribution was offered by 31% of the Proposers in the (5/5) game, and 73% in the (2/8) game. Thus, the Responders in Experiment 1a were offered the (8/2) distribution with the probability of 0.31 in the (5/5) game, and that of 0.73 in the (2/8) game. The participants played the games for real money, but currency was defined as Monetary Unit (MU), where 1 MU was equal to 0.5 AUD. The experiment was conducted and run with the Runtime Revolution Software.

#### Design

The Responders participated in all four Mini UGs presented in Table 1. They were asked to indicate their acceptance/rejection decisions for each of the two possible distributions in each game before hearing the actual distribution offered [see Falk et al. (2003a) for further information regarding this strategy method]. For example, in the (10/0) game, the Responders were asked whether they would accept or reject if the Proposer offered them the (10/0) distribution instead of (8/2); and they were subsequently asked whether they would accept or reject if the Proposer offered the (8/2) distribution instead of (10/0). If the game was (8/2), they were simply asked what they would do if the Proposer had no choice but to offer the (8/2) distribution. Once the Responders indicated their rejection/acceptance decision for each possible distribution, they simply moved on to the next game. After the completion of all four games, the Responders were informed about the overall outcomes and debriefed about the real set-up of the experiment (i.e., the offers were not made by actual proposers). The presentation order of the Mini UGs and that of the possible distributions in each game were randomized.

### Results

Table 2 shows the rejection rates of (8/2) distribution in different games. The main pattern observed in the previous studies (i.e., Falk et al., 2003a; Sutter, 2007) was replicated in our participant pool. To test the overall rejection rate differences across four games, we ran Cochran's *Q*-test. The test confirmed that the rejection rates of the (8/2) distribution were significantly different across four games (*p* < 0.0001). The rejection rate of the (8/2) distribution in the (5/5) game was the highest among four games. McNemar change tests were performed for the pairwise comparisons and they showed that the rejection rate in the (5/5) game was significantly higher than that of the (10/0) (*p* < 0.0001). The rejection rate of the (8/2) distribution was also significantly higher in the (5/5) game than in the (2/8) and the (8/2) games, *p* = 0.049, and *p* < 0.0001, respectively. In addition, the differences between the (2/8) and the (8/2) games, and the (2/8) and the (10/0) games were significant, *p* = 0.001 and *p* = 0.004, respectively. These results confirmed the previous findings that the rejections to an (unfair) offer were not determined by the absolute amount of money, but by how fair or unfair that offer was perceived in comparison to the other available offers^3^.

**Table 2:** **Rejection rates (in percentages) of (8/2) distribution across games in Experiment 1a (*N* = 50), 1b (*N* = 45), and 2 (*N* = 96)**.

	**Rejection rates of (8/2) distribution**			
	**(5/5)**	**(2/8)**	**(10/0)**	**(8/2)**
	**Game (%)**	**Game (%)**	**Game (%)**	**Game (%)**
Experiment 1a	60	42	18	14
Experiment 1b	37	33	15	22
Experiment 2								
R1	58	58	33	74
R2	62	21	25	42
R3	37	45	8	62
R4	50	30	25	17
Average	52	38	23	49

*R1, R2, R3, and R4 correspond to Round 1, Round 2, Round 3, and Round 4 of Experiment 2, respectively. Percentages reported for the row “average” are the rejection rates collapsed across rounds of Experiment 2*.

## Experiment 1B

Our main manipulation in this experiment was to eliminate the possibility of any attributions to intentions of the Proposer. To do so, the participants were informed that there were two distributions to be offered but that the actual offer would be determined by a random mechanism (Blount, 1995). Thus, the decision was not under the Proposer's full control, and therefore, it was impossible to evaluate the intentions behind the offer (Falk et al., 2008). When the fairness of intentions cannot be evaluated, the response should then only be determined by the outcome fairness if fairness considerations are the underlying force behind the Responder's responses. Thus, we expected that the rejection rates of the (8/2) distribution would still vary depending only on the relative fairness of the alternative outcomes but the differences across games should not be as large as they were in Experiment 1a [i.e., the rejection rate of the (8/2) distribution in the (5/5) game should not be as high as it was in Experiment 1 because now the alternative (5/5) offer was not intentionally ignored in the same way that the unequal (8/2) was not intentionally offered]. However, the rejection rates of the (8/2) distribution should still be the highest in the (5/5) game because the (5/5) distribution yields a more equitable *outcome* for each player.

### Method

Forty-five first year psychology students (*M* age = 19.8, 20 female) at UNSW participated in the experiment in return for a course credit. They were paid in accordance with the outcome of their decisions. The design and procedure of the experiment were almost the same as that of Experiment 1, with two exceptions. First, the participants were told that the offer of the Proposer would be determined by a coin flip [i.e., if it came up heads, the Proposer was going to offer the (8/2) distribution, otherwise the (5/5) distribution in the (5/5) game]. Second, the actual offer was indeed determined randomly [i.e., the (8/2) distribution was offered by the computer with probability of 0.5 in each game]. UNSW Human Research Ethics Advisory Panel approved the study.

### Results

The rejection rates of the (8/2) distribution across games are shown in Table 2. The rejection rates were only weakly different across four games, (*p* = 0.055, Cochran's *Q*-test). The only significant difference in terms of the rate of rejections for the (8/2) distribution within the games was between the (5/5) and the (10/0) games (*p* = 0.04, McNemar change tests)^4^. Cross-experimental analysis showed that the (8/2) distribution was rejected in the (5/5) game less frequently in Experiment 1b than in Experiment 1a, χ^2^ (1, *N* = 95) = 4.57, *p* = 0.033.

The results demonstrated that when the intentions of the Proposer were not assessable, (1) rejection rates of the (8/2) distribution significantly decreased when its alternative was a more equitable one [i.e., (5/5)], and (2) the overall difference in the rejection rates of the (8/2) distribution across four games was not strongly significant. Even then, however, the rejection rates were still not identical across the four games. This is an indication that these rejections were shaped by how unfair the outcome was perceived in comparison to the outcomes yielded by the alternative distributions. Overall, these results support the idea that two aspects of fairness considerations are involved in the emergence of costly punishment, but the contribution of the intention aspect of fairness considerations seems greater, especially when the alternative distribution yields a more equitable outcome [i.e., the (5/5) game].

## Experiment 2

In order to test the effect of the real possibility of RB and FI we changed the structure of the Mini UG from being one-shot and anonymously played to being iterated and non-anonymously played. We predicted that the rejection rates of the (8/2) distribution in the Mini UG should be (1) even higher (than in the one-shot, anonymous version) when its alternative was the (5/5) distribution because it is adaptive to build the reputation that one is a tough bargainer who rejects unfair offers, and (2) even lower when its alternative was the (10/0) distribution because it is adaptive to give the message for future interactions that one is capable to discern and will reward fair intentions.

### Method

#### Participants

One hundred and ninety-two first year psychology students (*M* age = 19.76, 120 female) at UNSW participated in the experiment as a part of their course requirement and were informed that they would be paid depending on the outcome of their choices. Four participants were tested in each experimental session and there were 48 experimental sessions^5^ in total. UNSW Human Research Ethics Advisory Panel approved the study.

#### Instructions phase

First, the participants were randomly allocated to their roles, (with 2 being Proposers, and the other 2 being Responders) and warned against revealing their allocated roles to the others. Individual players were then given detailed verbal instructions (along with a written instructions document) regarding the general structure of the game play, what their roles required them to do, and what the consequences of their accept/reject decisions would be. They were informed that they would play the game for more than one round with the same partner, and that their decision would be announced to other players right before they switched their partners. However, the players were not given any information about (1) the possible distributions available to the Proposer in Mini UGs [i.e., in order to eliminate the possibility of the (un)fairness of subsequent offers confounding the players' current decisions], (2) how many rounds they would play in total (i.e., in order to make the “shadow of the future” long enough), and (3) when exactly they would switch partners (i.e., in order to make the possibility of RB stronger). In order to eliminate a potential wealth effect, the participants were told that the overall amount that they would receive would be determined by a coin flip at the end of the experiment. If the coin toss came up heads, then they would get paid the amount that they earned in the first half of the experiment, and if tails, the amount earned in the second half (please see the Appendix section for the complete instructions). Afterwards, the instructions documents were collected, and the players were taken to the separate rooms to complete a short quiz (included in Appendix) measuring whether all the instructions were understood clearly.

#### Design

Each experimental session consisted of four consecutive rounds and in each round the participants played a different Mini UG game [i.e., the (5/5) game in Round 1, the (8/2) game in Round 2 and so on. Note that the allocation of the games into particular rounds was randomized]^6^. Each player was matched with his/her first game partner (i.e., Proposer 1 with Responder 1) before Round 1 and played two consecutive rounds (e.g., Round 1 and Round 2) with the same partner. After the completion of Round 2, they switched their partners (i.e., Proposer 1 started playing with Responder 2) and played the following two rounds (Round 3 and Round 4) with their new partners. At the end of each round, the decisions of both players (and the resulting outcomes) were announced to the players. These announcements were done privately (i.e., only between the pairs) after Round 1 and after Round 3; but publicly (i.e., to all players) after Round 2 and Round 4. For example, the decisions of Responder 1 and Proposer 1 were announced only to these two players after they completed Round 1, but their overall decisions in Round 1 and Round 2 were announced to all players just before they switched their partners.

#### Game play

In all Mini UGs, the Proposer was asked to choose one of the two available distributions (see Table 1). Simultaneously the Responder, without knowing what the Proposer had chosen to offer, was asked to indicate his/her acceptance/rejection decisions for each of the two possible distributions. (If the Responder had accepted the offer that the Proposer had chosen, the amount was distributed in accordance with the proposal. Otherwise, both got nothing). Both players were informed about the outcome right after the game was over, and then they moved on to the next game. The currency in the experiment was defined in MUs, where 1 MU equals 0.5 AUD. The experiment was conducted and run with *z*-Tree (Fischbacher, 2007). After the game play was over, both players received a questionnaire that was simultaneously prepared on the basis of the players' actual decisions in the experiment. The Proposers were asked to indicate why they offered the amount they offered and the Responders were asked for each game why they rejected/accepted the (8/2) distribution (please see the Appendix section for the respective questionnaires).

### Results

All participants passed the quiz distributed before the game play, thus all responses were included in the analysis. We first examined the extent to which the possibility of RB and FI influenced the Responders' overall rejection rates of the (8/2) distribution in each game in order to see how the opportunity of RB and FI could change this overall rejection pattern in each game. Table 2 (the bottom row) presents the rejection rates of the (8/2) distribution in different games, collapsed across rounds. The highest rejection rate was obtained in the (5/5) game and the lowest in the (10/0) game. These rejection rates of the (8/2) distribution were significantly different across four groups (*p* < 0.0001, Cochran's *Q*-test)^7^. Interestingly, almost half of the participants rejected the (8/2) distribution in the (8/2) game. McNemar change tests indicated that the rejection rate in the (5/5) game was significantly higher than that in the (10/0) and the (2/8) games, *p* < 0.0001, and *p* = 0.035, respectively, but not than those in the (8/2) game, *p* = 0.75^8^.

A cross-experimental comparison demonstrated that the rejection rates of the (8/2) distribution between Experiment 1a and Experiment 2 did not significantly differ in the (5/5) games [χ^2^(1, *N* = 146) = 0.83, *p* = 0.36], the (2/8) games [χ^2^(1, *N* = 146) = 0.16, *p* = 0.68], and the (10/0) games [χ^2^(1, *N* = 146) = 0.47, *p* = 0.49]. Contrary to our expectations, the rejection rates of the (8/2) distribution did not increase when the alternative distribution was (5/5), and did not decrease when the alternative distribution was (10/0). However, the (8/2) distribution was rejected in the (8/2) game much more frequently in Experiment 2 than Experiment 1a, χ^2^(1, *N* = 146) = 15.14, *p* = 0.0001. Similar patterns of differences were obtained in the comparison of Experiment 1b and Experiment 2. No significant differences were found between Experiment 1b and Experiment 2 in terms of the rejection rates of the (8/2) distribution in the (5/5) [χ^2^(1, *N* = 141) = 2.49, *p* = 0.11]^9^, the (2/8) [χ^2^(1, *N* = 141) = 0.36, *p* = 0.55], and the (10/0) games [χ^2^(1, *N* = 141) = 1.00, *p* = 0.32]. However, rejection rates for the (8/2) distribution were much higher in Experiment 2 than Experiment 1b in the (8/2) game, χ^2^(1, *N* = 141) = 8.61, *p* = 0.003. We will return to the interpretation of these results in the General Discussion^10^.

**Figure 1:**
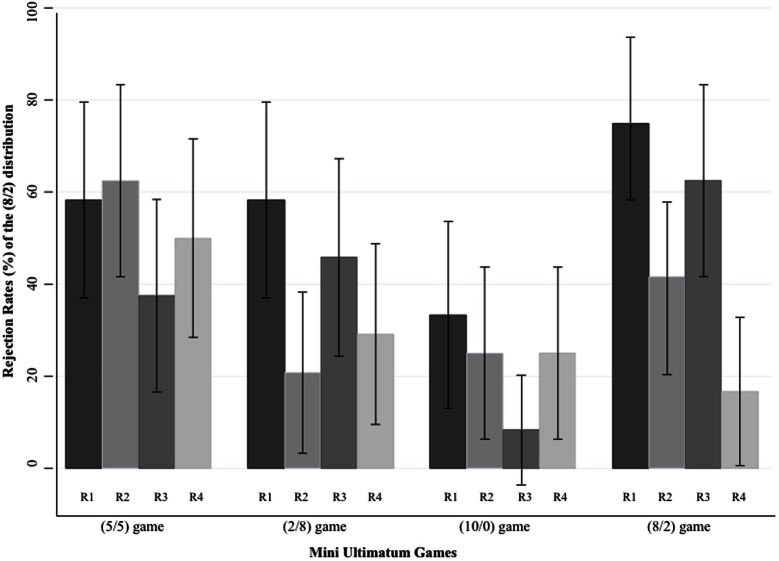
**Rejection rates of the (8/2) distribution across rounds in each game in Experiment 2.** Each bar in the figure represents percentage of Responders (out of 24) who rejected the (8/2) distribution in corresponding games. R1, R2, R3, and R4 correspond to Round 1, Round 2, Round 3, and Round 4, respectively. Error bars represent the 95% Confidence Interval.

We then investigated round by round rejection rates in all games of Experiment 2. The rationale of the round-wise analysis was (1) to investigate the reason behind the unexpectedly high levels of rejections in the (8/2) game, and (2) to see the effect of the possibility of RB and FI more clearly. We first focused on patterns (in rejections) indicating any type of signaling from the Responders to the Proposers, in terms of what Responders would not like to be offered. Even though it was possible to see the effect of RB and FI in all four rounds (i.e., because the players did not know how many rounds they would play in total nor when exactly they would switch partners, they should have incentive to build reputation for future interactions in all rounds), the rates of rejections of the (8/2) distribution were especially important in Round 1 and Round 3. Because the Responders would have a chance to give a message to their newly matched partners, they would (presumably) perceive these rounds (1 and 3) as the most suitable time to signal their preferences to their game partners for the following rounds. Figure 1 depicts the rejection rates (in percentages) of the (8/2) distribution across four rounds in each game of Experiment 2.

For the analysis of round by round rejection patterns, we first conducted a logistic regression by including dummy variables for different rounds, and correcting the standard errors for the clustering on participants (i.e., because we had independent samples for round-wise comparisons but matched samples for four Mini UGs). Afterwards, we tested the pairwise round differences for each game. Table 3 demonstrates the round by round differences in all games and both the across-rounds and the pairwise significance levels. For the (5/5) game, there was no significant differences across rounds in terms of the rejection rates of the (8/2) distribution [LR χ^2^(3, *N* = 96) = 3.39, *p* = 0.33]. A similar pattern obtained in the (10/0) game as well: except for the difference between Round 1 and Round 3 [LR χ^2^(1, *N* = 48) = 3.92, *p* = 0.05]. The rate of rejections in the (2/8) game in Round 1 was significantly higher than in Round 2 [LR χ^2^(1, *N* = 48) = 6.52, *p* = 0.01], but not in Round 3 than Round 4 [LR χ^2^(1, *N* = 48) = 1.39, *p* = 0.24]. However, the rejection pattern for the (8/2) game was different: the rejection rates of the (8/2) distribution were especially high in Round 1 and Round 3 (see Figure 1). The (8/2) distribution was rejected much more frequently in Round 1 than Round 2 [LR χ^2^(1, *N* = 48) = 5.18, *p* = 0.02], and in Round 3 than Round 4 [LR χ^2^(1, *N* = 48) = 9.31, *p* = 0.0002]. This pattern indicates that the effect of RB and FI was especially prevalent in the (8/2) game.

**Table 3:** **Significance levels (i.e., *p*-values) of across-round and pair-wise round differences in rejection rates of the (8/2) distribution obtained through logistic regression analysis for each Mini UG**.

**Significance levels of the differences (p-values) across rounds in Experiment 2**				
**Comparisons**	**(5/5) Game**	**(2/8) Game**	**(10/0) Game**	**(8/2) Game**
All rounds	0.33	0.05	0.27	0.001^*^
R1 vs. R2	0.76	0.01^*^	0.53	0.02^*^
R1 vs. R3	0.15	0.39	0.05	0.35
R1 vs. R4	0.56	0.04^*^	0.52	0.0002^*^
R2 vs. R3	0.08	0.08	0.14	0.15
R2 vs. R4	0.38	0.51	1.0	0.07
R3 vs. R4	0.39	0.24	0.14	0.0023^*^

For example, the intersection of the first column and the third row corresponds to the significance level of difference between Round 1 and Round 3 in terms of the rejection rates of the (8/2) distribution in the (5/5) game. The rejection rate was 58% in Round 1 and 37% in Round 3 in the (5/5) game (see Table 2), and these rates did not significantly differ, p = 0.15.

[*] * sign corresponds to significant differences (i.e., p < 0.05)*.

We also compared the round-wise rejection rates of the (8/2) distribution in each game in Experiment 2 with the rejection rates in the corresponding games in Experiment 1a and Experiment 1b (see Table 4 for complete lists of significance values revealed through comparisons of round-wise rejections in Experiment 2 with Experiment 1a and 1b for each game). Nevertheless, such analyses did not reveal anything different than the above-mentioned results demonstrating the null effect of RB and FI on rejections, except for the (8/2) game. For the (5/5) and the (10/0) games, the rejection rates of the (8/2) distribution in none of the rounds in Experiment 2 were significantly different than those in Experiment 1a and 1b. For the (2/8) game, only the rejection rate in Round 1 of Experiment 2 was marginally higher than that of Experiment 1b (*p* = 0.05). However, for the (8/2) game, rejection rates of the (8/2) distribution, especially in Round 1 and Round 3 were significantly higher in Experiment 2 than those in both Experiment 1a and 1b (see the last two columns of Table 4).

**Table 4:** **Significance levels (i.e., *p*-values) of differences in rejection rates of the (8/2) distribution obtained through the comparison of each round of Experiment 2 for each Mini UG with Experiment 1a and Experiment 1b for corresponding Mini UG**.

	**(5/5) Game**		**(2/8) Game**		**(10/0) Game**		**(8/2) Game**	
**Experiment 2**	**Experiment 1a**	**Experiment 1b**	**Experiment 1a**	**Experiment 1b**	**Experiment 1a**	**Experiment 1b**	**Experiment 1a**	**Experiment 1b**
R1	0.89	0.10	0.19	0.05^*^	0.15	0.09	0.00^*^	0.00^*^
R2	0.84	0.06	0.08	0.28	0.48	0.34	0.01^*^	0.09
R3	0.07	0.98	0.75	0.31	0.28	0.40	0.00^*^	0.00^*^
R4	0.42	0.33	0.29	0.72	0.48	0.34	0.76	0.58

For example, the intersection of the fourth column and the second row reads the significance level (0.28) of the difference between the rejection rate obtained in Round 2 of the (2/8) game in Experiment 2 (i.e., 21%—see Table 2) and the rejection rate obtained in the (2/8) game in Experiment 1b (i.e., 33%).

[*] * sign corresponds to significant differences (i.e., p < 0.05)*.

## General discussion

In Experiment 1a, we confirmed that people (negatively) respond to intentional unfairness in a Mini UG at a cost to their own material payoff. The difference in the pattern of results between Experiment 1a and 1b showed the relative impact of the aspects of the fairness consideration in shaping altruistically cooperative behaviors. We found that when a distribution yielding an unequal outcome between players was intentionally offered in the presence of a more equitable distribution [i.e., the (5/5) distribution], that distribution was rejected much more frequently (i.e., in Experiment 1, 60%) than when it was unintentionally offered (i.e., in Experiment 1b, 37%). This pattern of results indicates that from the perspective of the Responder, the intentions of the Proposer matter significantly. However, even when the intentions of the Proposers are not involved, another aspect of the fairness considerations is still present: it is the (un)fairness of the outcome distribution that governs the Responders' rejection behavior. The rejection rates of the unequal distribution were changed depending on the relative equitability of the alternative distribution.

In the literature, there are two distinct approaches to the fairness preferences over material benefits (Falk et al., 2008). These are the intention-based approach to the fairness concept (Rabin, 1993; Dufwenberg and Kirchsteiger, 2004) in which the emphasis is on the (fair/unfair) intentions behind an offer; and the outcome-based models (e.g., Fehr and Schmidt, 1999; Bolton and Ockenfels, 2000) in which the fairness is interpreted as the consideration of ending up with equitable material payoffs. Our results demonstrated that these two aspects of fairness considerations are differentially effective in determining the decisions of players. Thus, these findings provide convincing support for the idea that the economic models of preference for fairness that exclusively focus either on the intentions or on outcome fairness fail to capture altruistically cooperative behavior as a whole (Falk et al., 2008). Our results are rather compatible with the models that take both intentions and concerns for equitable outcomes into account (i.e., Falk and Fischbacher, 2006).

However, contrary to our predictions, the results of Experiment 2 indicated that the additional effect of the possibility of RB and FI did not lead to an increase in altruistic cooperation: rejection rates of the (8/2) distribution did not change when the Responders were expected to punish unfair offers (i.e., the 5/5 game) or to appreciate fair offers (i.e., the 10/0 game). Cross-experimental comparisons of the rejection rates obtained in Experiment 1a (i.e., involving both intention and outcome fairness) and Experiment 1b (i.e., involving outcome fairness only) with the overall rejections rates in Experiment 2 (i.e., involving RB and FI opportunity along with fairness considerations) confirmed that there were no changes in the rejections of an inequitable distribution in the (5/5) and the (10/0) games when the possibility of RB and FI was incorporated in to the context.

Two potential but competing explanations of this pattern of results can be offered. One is that the possibility of RB and FI is indeed (mis)perceived in one-shot and anonymously played games, and thus did not lead to any differences in the pattern of responses when it was explicitly incorporated into the context (Haley and Fessler, 2005; Bateson et al., 2006). The other is that the explicit incorporation of the possibility of RB and FI did not have any additional effect on the responses in the presence of the influence of fairness considerations (that are already effective enough to determine the rates of rejection). Unexpectedly high rejection rates of the (8/2) distribution observed in the (8/2) game in Experiment 2, as well as the round by round analyses of these rejection rates in each game strongly provide supporting evidence for the latter explanation.

The possibility of RB and FI led to an increase in the overall rejection rates only in a particular game where the intention of the Proposer was not assessable [the (8/2) game], but not in the other games in which the intentions were assessable [the (5/5), the (10/0), and the (2/8) games] (please see Table 2). This is the first indication of the effect of RB and FI being too weak to overcome the effect of fairness considerations. The Responders might only be taking the perceived intentions of the Proposers into consideration as a determinant of their accept/reject decisions for an unequal offer, and thus might not need to have additional reasons/concerns to change those decisions even when RB and FI are possible.

Round-wise analyses of the games in Experiment 2 support the claim that the possibility of RB and FI per se was not effective in changing the rejection responses, especially when the intentions were assessable: there was no variation across rounds in terms of the rejection rates of the (8/2) distribution, especially in the (5/5) and (10/0) games^11^. However, the effect of RB and FI did become effective once the fairness consideration is weakened as a result of the removal of the possible intentions behind an offer in the (8/2) game: it makes the Responders overly react against the unfairness of the outcome of the (8/2) distribution, most likely, in order to increase the possibility of being treated fairly in the future (Kiyonari et al., 2000; Hertel et al., 2002). This interpretation is mainly supported by the comparison of the rejection rates obtained in the (8/2) game across rounds in Experiment 2. The round-wise analysis of Experiment 2 (see Figure 1) showed that the rejection rates were significantly higher both in Round 1 (than Round 2) and Round 3 (than Round 4) only in the (8/2) game, where the intentions of the Proposer was not assessable. As stated previously, these two rounds were particularly important for the Responders to convey their message for future encounters. The implicit message given under such condition could be that they do not like to be offered an unequal distribution by *the same or the next game* partner in the following rounds. The Responders' self-reports collected after the game play also confirmed that the main purpose of the rejections in this game was indeed to tell the Proposers that “I will reject again if you ever propose such an unequal distribution.”

The results indicate that the absence of fairness intentions was the primary reason for the possibility of RB and FI becoming effective. However, the comparison between Experiment 1b and Experiment 2 revealed the importance of “outcome fairness” aspect of fairness considerations as well. This is because, except for the (8/2) game, the rejection rates of the (8/2) distribution obtained in none of the games were significantly different between Experiment 2 and Experiment 1b. This finding implies that even the presence of outcome fairness [i.e., perceived fairness of the (8/2) distribution relative to its alternative distribution] itself is strong enough to make the rejection rates reach a certain level—a level that could not get increased [i.e., for the (5/5) game] or decreased [i.e., for the (10/0) game] by the explicit incorporation of RB and FI. The possibility of RB and FI matters only when there is no intention behind the distribution offered, and there is no (more equitable or inequitable) alternative distribution to be offered [i.e., the (8/2) game].

These results shed light on when and how the possibility of RB and FI influence the responses in bargaining games. The possibility of RB and FI is normally expected to increase the rejections in the Mini UG as the Responders want to build the reputation of being a “tough bargainer.” This is why such opportunity has been sometimes thought to be the source of conflict (i.e., reduction in overall pay-off-wise efficiency) in strategic environments (Falk et al., 2003b). Our findings suggest that the possibility of RB and FI can lead to the respective conflict only when the fairness intentions are not assessable (i.e., the absence of the evaluation of intentions seems to increase the rejections in Mini UG, and thus leads to a “zero” outcome for both parties).

The current set of studies demonstrates the importance of fairness considerations, especially that of (un)fair intentions, in interactive economic decisions, particularly in the ultimatum bargaining games. The main conclusion drawn from the experiments reported here is that when both outcome and intention fairness considerations are involved in decisions they have a strong combined effect on the emergence of costly punishment as a form of altruistic cooperation, and thus override the potential influence of RB and FI. When an important aspect of fairness concerns, namely intentions, are absent, RB and FI may play an important role.

### Conflict of interest statement

The authors declare that the research was conducted in the absence of any commercial or financial relationships that could be construed as a potential conflict of interest.

## Footnotes

^1^We acknowledge that there are other forms of altruistic cooperation, such as cooperation in public goods games without any involvement of punishment of rewarding. However, the main interest of the current studies is costly punishment in Ultimatum bargaining games as a form of altruistic cooperation.

^2^We argue that rejections of *inequitable distributions* in UGs can be interpreted as costly punishment because rejection of any non-zero offer (even any unevenly distributed offer) is (1) costly to the Responder himself because as a results he ends up with a zero outcome, and (2) a form of punishment to the Proposer's unfairness as the Proposer also gets nothing when a rejection is made.

^3^The rejection rates for the alternative distributions (5/5), (2/8), and (10/0) were 2%, 6%, and 82%, respectively in Experiment 1a.

^4^The alternative distributions (5/5) and (2/8) were rejected by only 1 Responder in each game while the (10/0) distribution by 78% of the Responders in Experiment 1b.

^5^We ran these 48 sessions in two separate blocks of 24 sessions. The second block of 24 sessions was conducted after one of the anonymous referees asked us to collect more data for this experiment. The demographics of participants in the two testing blocks was very similar and participants were tested by the same experimenter in the same laboratory. The data from these two blocks of sessions were first analysed separately to check whether patterns of responding were the same across sessions. This was confirmed via statistical analysis showing no significant differences in overall rates of rejection across the two session blocks. Thus data from both sessions was combined for the analyses presented in the body of the paper. Please see Footnote 8 for the details regarding the results of statistical analysis.

^6^In order to eliminate a potential confounding sequence effect, we kept the round order of four Mini UGs different in each experimental session. Initially, there were 24 (i.e., 4! = 24) possible different orders, and thus we ran 24 sessions with a different sequence of Mini UGs in each. As we stated above, we later conducted another block with 24 experimental sessions as requested by one of our referees. For that reason, each of those 24 sequences had to be realized twice in total in the experiment.

^7^The rejection rates of the alternative distributions in the (5/5), (2/8), and (10/0) games were as follows: the (2/8) distribution was rejected by 3%, and the (5/5) distribution by 2%. Almost 91% rejected the (10/0) distribution.

^8^As stated in the Method section, we collected additional data from 96 participants (i.e., in an additional 24 sessions with four participants in each) in accordance with the suggestion of one of our anonymous referees. We ran additional statistical analyses in order to detect if there was any significant differences between these two blocks of 24 sessions (i.e., between the initial 24 sessions and the second 24 sessions). In particular, we compared these two separate blocks in terms of the overall rejection rates obtained in each Mini UG, and we found no significant differences. For the (5/5) game, the rejection rates were 52% vs. 52%, [χ^2^(1, *N* = 96) = 0.00, *p* = 1]; for the (2/8) game 41% vs. 35%, [χ^2^(1, *N* = 96) = 0.40, *p* = 0.53]; for the (10/0) game 18% vs. 27%, [χ^2^ (1, *N* = 96) = 0.94, *p* = 0.33]; and for the (8/2) game 50% vs. 48%, [χ^2^ (1, *N* = 96) = 0.04, *p* = 0.84] in the first block and second blocks, respectively.

^9^Note that the cross-experimental comparison between Experiment 1a and 1b demonstrated that the rejection rates of the (8/2) distribution in the (5/5) game were significantly lower when the intentions of the Proposer were not assessable. Thus, if the effect of RB and FI is dependent on the absence of intentions, then we would observe significantly higher rates of rejections in Experiment 2 as compared to Experiment 1b for the (5/5) game. Even though this expectation was met (i.e., rejection rates were 33% in Experiment 1b and 52% in Experiment 2), the respective difference was not statistically significant (*p* = 0.11), presumably due to the relatively low number of observations in Experiment 1b (*n* = 45).

^10^Due to the variation in proportion of female and male participants in all our experiments, we checked to see if there was any effect of gender in general, or gender by Mini UG interaction on rejection rates of the (8/2) distribution. Even though male participants tended to reject the (8/2) distribution more, there was no significant main effect of gender, *p* = 0.42, *p* = 0.35, *p* = 0.14 in Experiment 1a, 1b, and 2, respectively. Also, there was no interaction effect of gender and the type of Mini UG.

^11^Here one could argue that if the opportunity of RB and FI was effective only in the absence of intentions [i.e., in the (8/2) game], why then were the rejection rates in Round 1 marginally significantly higher than those in Round 2 in the (2/8) game—a pattern that indicates an effect of RB and FI in the presence of intentions. Note that the effect of RB and FI was still not strong in this particular game: First, the overall rejection responses for the (8/2) distribution did not differ with or without the involvement of RB and FI [compare the overall rejection rates in Experiment 1a, 1b, and 2 for the (2/8) game, rows 1st, 2nd, 8th in Table 2]. Second, the difference between Round 3 and Round 4 (see the last row of the 2nd column in Table 3) was not significant in terms of the rejection rates. This is a further indication that the effect of RB and FI was not strong.

## Appendix

### A. Instructions

#### Interactive decision making in economic games

Welcome and thank you for participating in this experiment. This experimental session consists of several parts. It's as follows:

1. Instructions for the experiment and assignment of the roles
2. Quiz
3. Actual experiment
4. Questionnaire

*(The experimenter asks participants to follow the written instructions while she is giving the instructions verbally)*

- In this study, you will be playing an economic game for several rounds.
- This is a two-person interactive game where “interactive” means each of you will be paired with a game partner.
- Since you are four participants here, there will be two groups containing two players in each.
- In this economic game, one of the players is the PROPOSER and the other one is the RESPONDER. So that means two of you will be playing as a Proposer and two of you as a Responder.

*(Assignment of the roles)*

- As you see, there are four cards on the table, each containing one role written on one side of each (i.e., Proposer 1, Proposer 2, Responder 1, Responder 2, respectively). I'll now turn the cards over, shuffle them and then you will pick one card randomly. Please do not reveal your role to the others afterwards. Note that you will keep these roles throughout the whole experiment.
- Rules of the game are as follows. Please listen carefully and make sure you understand what your role requires you to do. If you have any questions, please ask the experimenter immediately.

*(Rules of the game)*

- In the beginning of each round, the Proposer will be given an amount of money, say $10. Then the Proposer is asked to divide this amount between himself and the Responder in either of ONLY two ways. For example, the Proposer can choose an option where he gives $6 to the Responder and take $4 for himself, OR, he can choose the other option where he gives $3 to the Responder and take $7 for himself. The Proposer can choose either way but cannot invent a new way of distributing the money. After the Proposer chooses to offer one of the two ways of distribution, he waits for the Responder's decision.
- Meanwhile, the Responder, being aware of the Proposer has two possible ways of distributing the given amount, is asked to indicate his decisions for each possible way of distribution. For example, the Responder will indicate his/her acceptance/rejection decision both for the $6 offer, and for the $3 offer.
- If the Responder accepts the offer, the amount is distributed in accordance with the proposal. If the Responder rejects the offer, both the Proposer and the Responder gets nothing. For example, if the Responder accepts the $3 offer, he gets $3 and the Proposer gets $7. If the Responder rejects this offer, both the Proposer and the Responder gets 0 (zero).
- However, the Responder makes these decisions *before* he learns what the Proposer has actually offered. After the Responder makes his decisions, he learns the real offer. So, the Responders will either get the offered amount (if he has already accepted the offer) or nothing (if he has already rejected the offer).
- After both players learn the outcome of current round, they will move on to the next round (if the current round is not the last round).

*(Important details about the experimental procedure)*

- However, there are some other important rules that you must know in order to play the game properly.
- As you have been already told, there are several rounds in this experimental session. In each round, the structure of the game mentioned above will remain the same, but the possible ways of distribution (offers available to be offered!) will be different from round to round.
- Each group (consisting of one Proposer and one Responder) will be playing the same games at the same time. For example, every group will simultaneously play, say Game X, in Round 1 and Game Y in Round 2 etc.
- After you play *more than one round* with the same partner, you will switch partners at some point. For example, if you are a Proposer, you will be matched with the Responder of the other group, and be playing with this new game partner in the following rounds. Your previous partner will be matched and be playing with the Proposer of the other group in the following rounds as well.
- You will get notified just before you start playing with a new partner.
- Before you switch your partner, your previous decisions will be announced publicly (to all players in the experimental session). For example, if you are a Responder, whether you have accepted or rejected the previous offers will be announced to all players (the Proposer and the Responder in the other group, your game partner and you) at this announcement stage. That also means that your next game partner will know what you have done so far.
- Do not forget that you will be playing with real money (given by the experimenter). However, currency in the experiment is defined as Monetary Units (MUs). 1 MU equals $0.5 (AUD). For example, if you win 10 MUs, you will be paid $5 (AUD).
- In the beginning of each round, the Proposers will be endowed with a fixed amount of MUs.
- At the end of the experimental session, you will get paid in accordance with your earnings that you get either through the first half of the experiment, or through the last half. This will be determined by a coin-flip. For example, suppose that you've played 10 rounds in total. If the coin toss comes up heads, then you will receive the amount that you earn in the first five rounds, otherwise in the last five rounds.
- These are the instructions that you need for the experiment. Now you will take a quiz, which measures how well you understand these instructions.

### B. Quiz

Please answer to the following questions.

- How many groups are there in the experiment?
- What will happen if the Responder rejects the offered amount?
- What will happen if the Responder accepts the offered amount?
- Are you going to play only one Round or more?
  Yes ___ No ___
- 10 MUs equal to $ ___ (Please fill in the blank).
- Will your previous offers/decisions be announced to other players at some point during the experiment? If so, when?
  Yes ___ (Please indicate below when) No ___
- Are you going to play with the same game partner throughout the whole game? If not, what will happen?
  Yes ___ No ___ (Please indicate what will happen then)
- How many players will there be in each group?
- Will you get paid at the end of the experiment?
- If the Proposer offers 4 MUs out of 10 MUs to the Responder, and
  - •if the Responder accepts the offer, the Proposer takes ___ MUs, and the Responder gets ___ MUs.
  - •if the Responder rejects the offer, the Proposer takes ___ MUs, and the Responder gets ___ MUs.
- Do you know how many rounds in total you will play? If yes, please indicate how many.
  Yes ___ (Please indicate how many) No ___
- Were your roles randomly assigned? If not, how?
  Yes ___ No ___ (Please indicate how)

### C. Questionnaire for proposers

*(Former brackets were filled in with the actual offers by the Proposer, and the latter with the alternative offers in corresponding rounds)*

- In the first round, you offered […] MUs instead of […] MUs. Why? Please answer below.
- In the second round, you offered […] MUs instead of […] MUs. Why? Please answer below.
- In the third round, you offered […] MUs instead of […] MUs. Why? Please answer below.
- In the fourth round, you offered […] MUs instead of […] MUs. Why? Please answer below.

Please indicate your

Age:
Gender:

Thanks for your participation. Please see the experimenter for getting paid (and debriefing).

### D. Questionnaire for responders

*[Former brackets were filled in with the actual decisions (either accept or reject) by the Responder, and the latter with corresponding rounds in that particular session.]*

- Why did you [(reject)/(accept)] 2 MUs when its alternative was 5 MUs in the […] round? Please explain below.
- Why did you [(reject)/(accept)] 2 MUs when its alternative was 8 MUs in the […] round? Please explain below.
- Why did you [(reject)/(accept)] 2 MUs when its alternative was 0 MUs in the […] round? Please explain below.
- Why did you [(reject)/(accept)] 2 MUs when its alternative was 2 MUs in the […] round? Please explain below.

Please indicate your

Age:
Gender:

Thanks for your participation. Please see the experimenter for getting paid (and debriefing).
